# Attachment and Invasion of *Neisseria meningitidis* to Host Cells Is Related to Surface Hydrophobicity, Bacterial Cell Size and Capsule

**DOI:** 10.1371/journal.pone.0055798

**Published:** 2013-02-06

**Authors:** Stephanie N. Bartley, Yih-Ling Tzeng, Kathryn Heel, Chiang W. Lee, Shakeel Mowlaboccus, Torsten Seemann, Wei Lu, Ya-Hsun Lin, Catherine S. Ryan, Christopher Peacock, David S. Stephens, John K. Davies, Charlene M. Kahler

**Affiliations:** 1 School of Pathology and Laboratory Medicine, The University of Western Australia, Perth, Western Australia, Australia; 2 Veterans Affairs Medical Center, Atlanta, Georgia, United States of America; 3 Emory University School of Medicine, Atlanta, Georgia, United States of America; 4 Centre for Microscopy, Characterisation and Analysis, and Translational Cancer Pathology Laboratory, School of Pathology and Laboratory Medicine, The University of Western Australia, Perth, Western Australia, Australia; 5 Victorian Bioinformatics Consortium, Monash University, Melbourne, Victoria, Australia; 6 Department of Microbiology, Monash University, Melbourne, Victoria, Australia; University of Würzburg, Germany

## Abstract

We compared exemplar strains from two hypervirulent clonal complexes, strain NMB-CDC from ST-8/11 cc and strain MC58 from ST-32/269 cc, in host cell attachment and invasion. Strain NMB-CDC attached to and invaded host cells at a significantly greater frequency than strain MC58. Type IV pili retained the primary role for initial attachment to host cells for both isolates regardless of pilin class and glycosylation pattern. In strain MC58, the serogroup B capsule was the major inhibitory determinant affecting both bacterial attachment to and invasion of host cells. Removal of terminal sialylation of lipooligosaccharide (LOS) in the presence of capsule did not influence rates of attachment or invasion for strain MC58. However, removal of either serogroup B capsule or LOS sialylation in strain NMB-CDC increased bacterial attachment to host cells to the same extent. Although the level of inhibition of attachment by capsule was different between these strains, the regulation of the capsule synthesis locus by the two-component response regulator MisR, and the level of surface capsule determined by flow cytometry were not significantly different. However, the diplococci of strain NMB-CDC were shown to have a 1.89-fold greater surface area than strain MC58 by flow cytometry. It was proposed that the increase in surface area without changing the amount of anchored glycolipid capsule in the outer membrane would result in a sparser capsule and increase surface hydrophobicity. Strain NMB-CDC was shown to be more hydrophobic than strain MC58 using hydrophobicity interaction chromatography and microbial adhesion-to-solvents assays. In conclusion, improved levels of adherence of strain NMB-CDC to cell lines was associated with increased bacterial cell surface and surface hydrophobicity. This study shows that there is diversity in bacterial cell surface area and surface hydrophobicity within *N. meningitidis* which influence steps in meningococcal pathogenesis.

## Introduction

*Neisseria meningitidis* is a Gram-negative diplococcus which is asymptomatically carried in the nasopharynx by approximately 10% of the adult population but is also the causative agent of epidemic septicaemia and meningitis which results in 5–20% case fatality rates [1]. Strains of *N. meningitidis* isolated from carriage are usually unencapsulated while invasive isolates are encapsulated, with the identity of the polymer being the foundation of serogroups [2], [3]. Based on the sequence similarity of housekeeping genes, *N. meningitidis* can be arranged into thirty-seven lineages of closely related sequence types (STs) termed clonal complexes (cc) which account for 61% of all strains collected [4]. Some of these clonal complexes are more often associated with carriage than disease (>2:1), while other clonal complexes have been deemed to be hyperinvasive as these strains are more commonly associated with invasive disease than carriage (generally >5:1) [5]. Within the hyperinvasive lineages, distinct phylogenetic clades (PC) are evident for ST-8/11 cc, ST-32/269 cc, and ST-41/44 cc [6]. Despite the existence of carriage and hyperinvasive clonal complexes, the only common virulence determinants for invasiveness of disease causing isolates is encapsulation [7] and the carriage of phages [8].

Representative strains of the ST-4, ST-18 and ST-32 hyperinvasive lineages have been used to examine the interaction of meningococci with host cells [9], [10], [11], [12], [13], [14], [15], [16]. The initial interaction between the meningococcus and the host cell is mediated through the meningococcal type IV pilus. The meningococcus then retracts the pilus bringing the bacterium into close contact with the host cell surface [17]. Intimate association of the meningococcus with the host cell membrane occurs through the binding by the meningococcal outer-membrane ***opa***city (Opa) and Opc adhesins to ***c***arcino***e***mbryonic ***a***ntigen ***c***ell ***a***dhesion ***m***olecules (CEACAMs), and to heparin sulfate proteoglycans and integrins through bridges of vitronectin and fibronectin [17]. The attachment and invasion of host cells is inhibited *in vitro* by the expression of capsule and lacto-*N*-neotetraose (LNT) α-chain on lipooligosaccharide (LOS) [18], [19]. The presence of capsule and LNT-bearing LOS both inhibit Opa function, presumably by physical occlusion or by acting as a ligand of Opa [18], [19]. Therefore meningococcal invasion of epithelial cells is favoured when LNT and capsule expression has been phased off through translational frame-shift due to the expansion or contraction of polymeric nucleotide tracts within the relevant biosynthetic genes. Since these virulence determinants play various and sometimes opposing roles in the infection process, phase variable expression of virulence determinants such as pili, LNT-bearing LOS and capsule is essential for the success of meningococcal pathogenesis [20].

The prototype strain of ST-8 cc, NMB-CDC [21], has been extensively characterised with regards to LOS structure, capsule expression [22], [23], [24], [25], [26], [27], [28], [29], [30], [31], [32], interaction with human monocytes [33], pilin [34], biofilm formation [35] and the rate of phase variation [36], as has the prototype ST-32 cc strain MC58 [12], [15], [16], [19], [37], [38], [39], [40], [41], [42], [43], [44]. Both isolates express a serogroup B capsule which is a membrane anchored glycolipid, consisting of a sugar polymer with an average length of approximately 300 α2→8 linked sialic acid residues covalently bound to a diacylglycerol lipid anchor which tethers the polymer to the outer membrane [22], [23], [24], [25], [26], [27], [28], [29], [30], [31], [32]. The sugar polymer is assembled at the cytoplasmic surface of the inner membrane, is moved to the lipid anchor and is translocated across the membranes *en bloc* to the bacterial cell surface [22], [23], [24], [25], [26], [27], [28], [29], [30], [31], [32]. However, strains NMB-CDC and MC58 express different LOS structures [45] and type IV pilin classes [34]. Strain MC58 expresses class I pili which undergo phase and antigenic variation [36], [46], while strain NMB-CDC expresses class II pili which do not undergo antigenic variation [36], [46], a phenotypic characteristic of strains from the ST-8/11 cc [6], [34], [47]. Since the model for meningococcal attachment and invasion of host cells relies on phase variation for this process to succeed [48] and since strain NMB-CDC is known to phase vary the expression of genes at low frequency [36], we compared prototype strain NMB-CDC to the ST-32 prototype strain MC58. In testing the role of known virulence determinants that affect meningococcal attachment and invasion into host cells, we also tested whether bacterial cell size differed between isolates. Dalia and Weiser [49] have recently shown that minimization of bacterial cell size allows for complement evasion by *Streptococcus pneumoniae* and has proposed that long chain variants may be suited to a mucosal lifestyle, with invasive strains typically retaining a diplococcal form. Therefore, we examined whether meningococcal isolates varied in surface area and looked for correlates with the rates of attachment and invasion.

## Methods

### Bacterial strains and growth conditions

*N. meningitidis* strains NMB-CDC (ST-8, serogroup B, immunotype L2,4, PilEII), and MC58 (ST-32, serogroup B, immunotype L3,7,9, PilEI) were cultured under aerobic conditions with 5% CO_2_ at 37°C on GC agar (GCA) or GC broth (GCB) (Oxoid) supplemented with 0.4% glucose, 0.01% glutamine, 0.2 mg of cocarboxylase per litre, and 5 mg of Fe(NO_3_)_3_ per litre. The wild-type strains and constructed mutants were piliated and expressed Opa, except where stated otherwise (Table 1). Antibiotic selection for meningococcal mutants was performed on GCA containing 100 µg/ml of kanamycin (sulfate salt), 60 µg/ml of spectinomycin, 5 µg/ml of tetracycline or 2 µg/ml of erythromycin (Sigma). *Escherichia coli* DH5α was used as a host for all DNA manipulations. It was routinely grown in Lysogeny broth (LB) or on Lysogeny agar (LA, Oxoid) which, where appropriate, were supplemented with antibiotics at the following concentrations: ampicillin at 100 µg/ml, spectinomycin at 50 µg/ml, kanamycin at 50 µg/ml, erythromycin at 300 µg/ml, tetracycline at 12.5 µg/ml and chloramphenicol at 30 µg/ml (Sigma).

**Table 1:** Strains used in this study.

Strain Name	Genotype	*Major Phenotype	Ref
NMB	B:2B:P1.2,5:L2 (CDC8201085)	Cap+, Opa+, NANA+LNT+, PilEII+, GLY+	[55]
MC58	B:15:P1.7,16b:L3,7,9 (ATCCBAA-335)	Cap+, Opa+, NANA+LNT+, PilEI+, GLY+	[95]
M7	NMBΔ*synA*::Tn916	Cap−, Opa+, NANA−LNT+, PilEII+, GLY+	[55]
JKD5106	NMBΔ*misR*::*tetM*	Cap+, Opa+, NANA+LNT+, PilEII+, GLY+	[29]
JKD5114	NMBΔ*pglF*::*aphA-3*	Cap+, Opa+, NANA+LNT+, PilEII+, GLY−	[34]
JKD5122	NMBΔ*pilE*::*aadA*	Cap+, Opa+, NANA+LNT+, PilEII−, GLY+	[34]
CKNM367	NMBΔ*synD*::*aphA-3*	Cap−, Opa+, NANA+LNT+, PilEII+, GLY+	This study
CKNM385	NMBΔ*lst*::*aphA-3*	Cap+, Opa+, NANA−LNT+, PilEII+, GLY+	This study
CKNM389	NMBΔ*pilE*::*ermC*	Cap+, Opa+, NANA+LNT+, PilEII−, GLY+	This study
CKNM390	NMBΔ*iga*::*pilEI*(MC58)-*aadA*	Cap+, Opa+, NANA+LNT+, PilEI+, PilEII+, GLY+	This study
CKNM394	NMBΔ*iga*::*aadA*	Cap+, Opa+, NANA+LNT+, PilEII+, GLY+	This study
CKNM396	NMBΔ*iga*::*aadA*Δ*pilE*::*ermC*	Cap+, Opa+, NANA+LNT+, PilEII−, GLY+	This study
CKNM397	NMBΔ*iga*::*pilEI*(MC58)-*aadA*Δ*pilEII*::*ermC*	Cap+, Opa+, NANA+LNT+, PilEI+, GLY+	This study
CKNM416	MC58Δ*pglF*::*aphA-3*	Cap+, Opa+, NANA+LNT+, PilEI+, GLY−	This study
CKNM417	MC58Δ*pilE*::*aphA-3*	Cap+, Opa+, NANA+LNT+, PilEI−, GLY+	This study
CKNM419	MC58Δ*synD*::*aphA-3*	Cap−, Opa+, NANA+LNT+, PilEI+, GLY+	This study
CKNM420	MC58Δ*synB*::*aadA*	Cap−, Opa+, NANA−LNT+, PilEI+, GLY+	This study
CKNM421	MC58Δ*lst*::*aphA-3*	Cap+, Opa+, NANA−LNT+, PilEI+, GLY+	This study
CKNM423	MC58Δ*misR*::*tetM*	Cap+, Opa+, NANA+LNT+, PilEI+, GLY+	This study

* Phenotypes: Capsule (Cap), Opacity proteins (Opa), *N*-acetyl neuraminic acid present on LOS (NANA), LOS α-chain of lacto-*N*-neotetraose (LNT), pilus expression (PilE), pilus class I or II (PilEI or PilEII), and the presence of glycosylation (GLY) which can occur on outer membrane proteins other than pilin.

### Genome sequencing, assembly and annotation

Sequencing of strain NMB-CDC was performed on a Roche 454 GS FLX and an Illumina GAIIx. The 454 yielded 92 Mbp (44×) from a 3 Kbp mate pair library, average read length 403 bp. The GAIIx yielded 370 Mbp (176×) of 36 bp paired-end reads from a 200 bp fragment library. *De novo* assembly of the 454 reads was performed using gsAssembler 2.6.0, resulting in 158 contigs longer than 200 bp within 5 scaffolds totaling 2.1 Mbp. The GAIIx reads were aligned to the 454 assembly to correct two 454 homopolymer assembly errors using Nesoni 0.63 (http://vicbioinformatics.com). The draft genome was annotated using Prokka 1.01 (http://vicbioinformatics.com). Prokka uses Prodigal, Aragorn and RNAmmer to identify CDS, tRNA, and rRNA features respectively. Gene function was predicted via HMMER3 profile searches against PFAM and CDD; and BLAST+ similarity searches against UniProtKB. All annotated protein sequences from published *Neisseria* genomes were clustered using OrthoMCL [50] with a cut-off of 1e −50 to identify any new genes. Venn diagrams were created using this website tool (http://creately.com/Draw-Venn-Diagrams-Online). GenBank Accession numbers for the genomes of the strains analysed in this study are as follows: M6190 (AEQF00000000.1) and ES14902 (AEQI00000000.1), G2136 (CP002419.1), FAM18 (NC_008767.1), MC58 (NC_003112.2), 961−5945 (AEQK00000000.1), S0108 (ADWN00000000.1), K1207 (ADWM00000000.1) and H44/76 (CP002420.1).

The *opaA* allele was amplified with primer pair KAP260 (5′-TACGCTGCAGAAAATGAATCCAGCCCCC-3′) and KAP261 (5′-ACATCGGAAATCCAAGTGTGTTCC-3′), to generate a 3 kb amplicon. The *opaB* allele was amplified with primer pair KAP230 (5′-AGGAGCAGTTCGCCTTGAGGG-3′) and KAP231 (5′-CAGACACCTGCAAGTATCCGC-3′), generating a 3 kb amplicon. The *opaD* allele was amplified with primer pair KAP232 (5′-TAACGGGTAGGGTATCGGTCGG-3′) and KAP233 (5′-TGGAACCCAAATCGACGGAGGC-3′), generating a 5 kb amplicon. The *opaJ* allele was amplified with primer pair KAP234 (5′-ACAACTTGGGCTTCCGTTGCACAAGC-3′) and KAP235 (5′-ACGCCGACAGAGGAAGTGCATAAGC-3′), resulting in a 4 kb amplicon. Each amplicon was used as a template for the PCR using primers KAP236 (5′-CCGTATTATGTGCAGGCGGATTTAGC-3′) and KAP237 (5′-TCCAAGCGTCCCCAGTTGTGG-3′) to confirm the presence of an *opa* allele in each locus, followed by sequencing with KAP236, KAP237 and KAP238 (5′-AATCGTGGGTAATGCGTTCGGC-3′) to identify both the sequence of the allele and the phase status. The *Neisseria meningitidis* Opa sequence database (http://neisseria.org/nm/typing/opa/) developed by Keith Jolley and Martin Callaghan and sited at the University of Oxford was used to identify the alleles.

The absence of *pilC2* from the genome sequence of NMB-CDC was noted. The density of the reads of the *pilC1* and *pilC2* loci were assessed and indicated that a single copy of *pilC* was present in the genome. As both loci appeared to have good coverage, we predicted that *pilC2* was absent from the genome of NMB-CDC. This was confirmed by PCR analysis using primer pair KAP509 (5′-CGTTTGTGGACGCACTGCTGG-3′) and KAP510 (5′-CGACAAATTCCGCACAGGCAGC-3′) which resulted in an amplicon of 2.3 kb when amplified from NMB-CDC, compared to an amplicon of 8.7 kb when amplified from MC58 which possesses the *pilC2* allele.

### Cell lines and culture

The immortalised Detroit 562 (human pharyngeal carcinoma epithelial cells, ATCC CCL-138) and 16HBE14σ- (transformed human bronchial epithelial cells) [35] cell lines were grown to confluence in Minimal Essential Media (MEM) with Earles salts supplemented with 10% fetal calf serum (FCS), 2 mM L-glutamine, 1 mM sodium pyruvate and 1× non-essential amino acids (Invitrogen) as per ATCC recommendations [51]. At confluence the cells were lifted with 0.05% trypsin/EDTA (Invitrogen) and resuspended in cell media. Cells were counted and 1×10^5^ cells were seeded into each of the wells of a 24-well plate (Nunc).

### Construction of mutants of strain NMB-CDC and MC58

The plasmids constructed and used in this study are listed in Table 2. To create capsule negative variants of strain NMB-CDC and MC58, *synD* was inactivated with a *synD::aphA-3* cassette. A region containing the 5′ end of *synD* was amplified from NMB-CDC gDNA with primer pair, KAP196 (5′- TATGCTTGCTTAGGAGCAGTAGC-3′) and KAP366 (5′-GATCTGCAGGATCCTAGCTGATTGATGAACTAACTTAGGC-3′). A region containing the 3′end of *synD* was amplified from NMB-CDC gDNA with primer pair, KAP369 (5′-CCTTGCAGCACATCCCCCTTTCG-3′) and KAP367 (5′-CTAGGATCCTGCAGATCTATTTGAGCTTCCTAGAAGCCC-3′). These first round PCR products were used as templates for a second round of PCR amplification with the nested primer pair KAP365 (5′-CTGCTTGCAATATTCGCAAAGGTGC-3′) and KAP368 (5′-GCATATTCAGGAAAGGGGACATGC-3′) to create a fusion product with internal *Bam*HI and *Pst*I sites introduced by KAP366 and KAP367. This fragment was cloned into the *Bam*HI and *Pst*I sites of pHSG576 which were removed by treatment with T4 DNA polymerase, resulting in pCMK718. The *aphA-3* cassette conferring kanamycin resistance was amplified from pUC18K with primer pair KAP128 and KAP129 and cloned into the *Bam*HI site of pCMK718 resulting in pCMK727. Strain NMB-CDC and strain MC58 were transformed with pCMK727 and the transformants were identified by resistance to kanamycin then confirmed by PCR using primer pair KAP196 and KAP369, resulting in CKNM367 and CKNM419, respectively.

**Table 2:** Plasmids used in this study.

Plasmid Name	Description	Reference
pAErmC'G	High copy vector carrying the *ermC'G* cassette	[96]
pHP45Ω	pUC18 carrying the *aadA* spectinomycin resistance determinant flanked by inverted repeats	[97]
pHSG298	Kanamycin resistant high copy cloning vector	[98]
pHSG576	Chloramphenicol resistant low copy cloning vector	[98]
pUC18K	pUC18 carrying *aphA-3* kanamycin resistance determinant forming a promoterless non-polar cassette	[99]
pCK90	pHSG576+*lst*	[27]
pCMK716	pHSG576+*pilEI*(MC58)	This study
pCMK718	pHSG576+*synD*	This study
pCMK720	pHSG576+*synB*	This study
pCMK723	pHSG576+*synB*::*aphA-3*	This study
pCMK727	pHSG576+*synD*::*aphA-3*	This study
pCMK737	pHSG576+*lst*::*aphA-3*	This study
pCMK739	pHSG576+*pilEI*(MC58)::*aphA-3*	This study
pJKD2407	pHSG576+*pglF*::*aphA-3*	[34]
pJKD2425	pHSG298+*pilEII*(NMB)	[34]
pJKD2426	pHSG298+*pilEII*(NMB)::*aadA*	[34]
pJKD2539	pHSG298+*misR*::*tetM*	[29]
pJKD2580	pHSG576+NMB*iga*	This study
pJKD2581	pHSG576+NMB*iga*::*aadA*	This study
pJKD2728	pHSG298+*pilEII*(NMB)::*ermC*	This study
pJKD3172	pUC18+MS11*iga::aadA*	C.S. Ryan
pJKD3341	pUC18+MS11*iga*::*pilEI*(MC58)-*aadA*Δ*pilEII*::*ermC*	This study

To construct an isolate of strain MC58 that does not synthesise sialic acid, the sialic acid synthetase, *synB*, was inactivated using a *synB*::*aadA* cassette. An internal region of *synB* was amplified from NMB-CDC gDNA with primer pair KAP371 (5′-GTTATACTTGCGCGCCAAAACTCC-3′) and KAP372 (5′-GTTCGTGGTTGTAACCTACTGAACG-3′). This fragment was cloned into the *Hin*dIII site of pHSG576, resulting in pCMK720. The cassette containing the spectinomycin resistance gene *aadA* was obtained from pHP45Ω by digestion with *Hin*dIII and cloned into the *Hin*dIII site of pCMK720 resulting in pCMK723. Strain MC58 was transformed with pCMK723 and the transformants were identified by resistance to spectinomycin then confirmed by PCR using primer pair KAP370 (5′-TCCTGAAACGTGGAATGTTTCTGC-3′) and KAP373 (5′-ATAGAGACATCTGCATTGCCTGG-3′), resulting in CKNM420.

To create mutants in which LOS sialylation was absent, *lst*, encoding the LOS sialylation transferase, was inactivated using a *lst*::*aphA-3* cassette. The *aphA-3* cassette conferring kanamycin resistance was amplified from pUC18K with primers KAP128 and KAP129 and cloned into the *Hin*cII site of pCK90 (Table 2) resulting in pCMK737. Strains NMB-CDC and MC58 were transformed with pCMK737 and the transformants were identified by resistance to kanamycin, then confirmed by PCR using primer pair KAP183 (5′- CAAAAGCCTGCACAATCGGCAGC-3′) and KAP184 (5′- GCAAATCCTGCCACGACAGTTTCC-3′), resulting in CKNM385 and CKNM421, respectively.

A mutant of strain MC58 that contained an inactivated *misR* was created by transformation with pJKD2539 (Table 2) to create CKNM423 as described previously [29].

### Transformation protocols

Strain NMB-CDC was transformed via natural transformation as described previously [52]. Natural transformation rates of strain MC58 are particularly low, therefore a modified chemical transformation procedure was used [53]. Approximately 2×10^9^ cfu were harvested after 12 hr growth on GCA and resuspended in 1 ml ice cold GC-TSB (transformation broth as previously described [54] made with GCB instead of LB). An aliquot of 200 µl was added to 200 ng of plasmid DNA and incubated on ice for 20 mins. A 1 ml aliquot of room temperature GCB was added to the bacteria/DNA mixture and incubated at 37°C with shaking for 1 hr. The bacteria were harvested via centrifugation and plated on GCA containing the appropriate antibiotics for the selection of transformants.

### Attachment and invasion assays

Detroit 562 epithelial cells and 16HBE14σ- cells were grown to confluence in 24-well plates and utilised for invasion assays. Invasion assays were conducted as described previously [40] with the following modifications. The epithelial cells were inoculated with suspensions of meningococci at a multiplicity of infection (MOI) of 100:1 in epithelial cell media containing 2% FCS [16]. The number of eukaryotic cells present in each well was estimated by sacrificing two wells for counting. To do this, the media was removed, the cell monolayer was washed with Dulbecco's PBS and the cells lifted with trypsin. The cells were enumerated using a haemocytometer and dead cells were excluded from this count by trypan blue staining. The average of the number of cells was used to estimate the number of eukaryotic cells present in each of the experimental wells for each experiment. Bacteria were harvested from a 14 hr agar plate culture, resuspended in GC broth and normalised to an OD560 nm of 0.4. In the original experiment, the viable count was determined for OD560 nm of 0.4. This standard was applied to each experiment thereafter to calculate the MOI of 100:1. For each experiment the actual number of bacteria was confirmed by viable count of the inoculum.

The epithelial cell monolayers were challenged with strains of *N. meningitidis* for either 1 hr or 6 hrs as indicated, and incubated in 5% CO_2_ at 37°C. Non-adherent bacteria were then removed by washing the monolayers three times with Dulbecco's PBS (Invitrogen). Following 1 hr incubation in either epithelial cell media containing 2% FCS (for enumeration of cell associated bacteria) or epithelial cell media containing 2% FCS with 100 µg/ml gentamycin (Sigma) (for enumeration of intracellular, protected bacteria), the monolayers were washed three times with Dulbecco's PBS to remove gentamycin and the epithelial cells were lysed with 1% saponin (Sigma) in epithelial cell media containing 2% FCS to release the intracellular bacteria. The number of cell associated bacteria and intracellular bacteria were enumerated by viable count. At least three biological repeats containing three technical repeats were performed for each strain. Attachment was determined as the proportion of the inoculum which had attached to epithelial cells, and invasion as the proportion of the attached bacteria which had invaded the epithelial cells. A Mann-Whitney t-test was used to determine statistically significant differences. All strains assayed were sensitive to gentamycin and resistant to 1% saponin as determined by viable count (data not shown).

### Determination of capsule, LOS, pilin and Opa phenotype

Colony immunoblotting was utilised to identify transformants expressing class I pilin and to determine the rate of phase variation of *synD* and *lgtA* as described previously [55]. Briefly, strains were plated at a dilution that obtained 30–300 colonies per GCA plate. The colonies were lifted using a nitrocellulose membrane (GE Healthcare) and the plates were re-incubated to allow the colonies to re-grow. The nitrocellulose discs were air dried, then fixed by UV exposure. The membranes were washed with TBS, then blocked with 2% BSA (Millipore) in TBS for 1 hr. Primary antibodies against class I pilin (MAb SM1 [56]), capsule (MAb 2-2-B [57]) and LNT (MAb 3F11 [58]) were used at concentrations of 1:100, 1:1000 and 1:10 in blocking buffer, respectively. Conjugated secondary antibodies (Santa Cruz Biotechnology) were used at a concentration of 1:1000 and the immunoblots were developed using NBT/BCIP was used as per the manufacturer's instructions (Sigma Cat#N6876 and Cat#B8503 respectively). A negative control strain was included in each assay.

Opa expression was determined by immunoblotting. Whole cell lysates (750 ng) were separated by 15% sodium dodecyl sulfate-polyacrylamide gel electrophoresis (SDS-PAGE) by standard methods and transferred to nitrocellulose membranes. The membranes were blocked overnight with 2% BSA in TBS. The polyclonal rabbit anti-Opa primary antibody [59], was used at 1:1,000. A horse radish peroxidase-conjugated anti-rabbit IgG secondary antibody at a dilution of 1:1000 (Santa Cruz Biotechnology) was used and the membrane was developed with an ECL kit (GE Healthcare).

### qRT-PCR

Total RNA was purified from bacteria using the High Pure RNA extraction kit (Roche). These RNA preparations were assayed for DNA contamination by PCR and nucleic acid concentration was determined by absorbance in a Nanodrop spectrophotometer (Thermo Scientific). cDNA was generated from 1 µg of total RNA as template and random hexamers using reverse transcriptase (New England Biolabs). A mock reaction lacking reverse transcriptase was also included and served as the negative control in the qRT-PCR reaction. qRT-PCR was performed using SYBR green I mastermix (Roche). Melt curve analysis confirmed the presence of a single product from each PCR reaction and the reverse transcriptase negative control was used to detect DNA contamination. The primer pairs used to assess the transcript levels were: *lpxA* were DAP135 (5′-CGTTTTGGGCGGCTACAC-3′) and DAP136 (5′-GGCGGTCATGGCGTAGTC-3′); *synA* were KAP353 (5′-GGTCATGATTCACGGCGAC-3′) and KAP354 (5′-CCTACAGCTGCGCCTGCTAG-3′); *ctrA* were KAP355 (5′-GTTTGGCGATGGTTAATGCTT-3′) and KAP356 (5′-ACCAACTGCTCTGGCAACTT-3′); *lst* were KAP343 (5′-CAGGTATTTGGGCGATGAGT-3′) and KAP344 (5′-GCCGTCGTCCATAATGTTTT-3′); *lgtG* were KAP333 (5′-GCTCGCTCAAACCAGAAAAA-3′) and KAP334 (5′-GGAATGCGCCATAATCAGTT-3′); and *dsbD* were KAP357 (5′-GTTGGGACAGCCTTCTTTCA-3′) and KAP358 (5′-TTGCATAAGGAAAGGCAACC-3′). The relative changes in gene transcription were calculated using the comparative C_T_ method [60], normalised to the level of *lpxA* transcript. Each set of qRT-PCRs was examined in triplicate and was repeated with at least three independent RNA preparations [61].

### Flow cytometry for the detection of meningococcal capsule and bacterial cell size using high throughput microscopy

The procedure for the detection of capsule by flow cytometry was essentially that of Tzeng *et al.* [31]. Meningococci grown to early stationary phase were collected and suspended in PBS. Cells were washed with 0.5 ml of PBSB buffer (0.5% [w/v] bovine serum albumin in PBS) once before the incubation with 100 µl of MAb 2-2-B (1:500 dilution) for 1 h at 37°C. After washing with 0.5 ml of PBSB buffer, the cells were further incubated with a 1:100 dilution of AF647-goat anti-mouse IgM (Invitrogen) for 1 hr at 37°C. Cell pellets were obtained after centrifugation and were resuspended in 0.5 ml of PBS and 0.5 ml of 5 µM SYTOX Green (Invitrogen), prepared in PBS from a 5 mM stock solution in dimethyl sulfoxide, to assess the membrane integrity of the bacteria [62], [63]. The cells were then analysed for relative size and fluorescence labelling with a BD Influx cell sorter (BD Biosciences), in a biohazard containment hood, utilising a 488 nm excitation laser and 520/15 nm emission filter for detection of SYTOX Green staining and 640 nm excitation laser and 670/30 nm emission filter for detection of the AF647 secondary antibody. All fluorescence emissions were acquired in log, and data was collected for 50,000 cells using BD Sortware V1.0.0.653 (BD Biosciences), the operating software on the BD Influx. Experimental data were analysed and geometric mean fluorescence was calculated using FlowJo V7.2.5 (Tree Star Inc.) flow cytometry analysis software. The surface area for spheres was calculated using: 4πr^2^ (where “r” is the radius) and multiplied by a factor of two for a diplococcus. For high throughput microscopy to determine cell size, ethanol fixed samples were prepared and stained with SYTOX Green as above. Images for 50,000 events were collected for each sample using the AMNIS ImageStream^x^ Imaging Cytometer (AMNIS Corporation) with INSPIRE V4.1 acquisition software. The bright field image was collected in channel 1 after white light illumination and the corresponding SYTOX Green signal collected in channel 2 (480–560 nm) after 488 nm laser excitation [64]. Out of focus cells, debris and cell clumps were excluded from statistical analysis and cell area calculated using IDEAS V5.0 image analysis software (AMNIS). Cell area was calculated using an erode mask on the bright field image to separate the image pixels from the background pixel intensity. Pixel number is then converted to area in µm^2^.

### Electrophoretic mobility shift assay (EMSA)

EMSA experiments were performed following previously reported procedures [61]. The promoter fragment was obtained by PCR using primer pair LJ6 (5′-CATCCTACAATTAAACTTCCACAC-3′) and JS44 (5′-GCTTGTTCATTTGCTACCAAGTGG-3′) and end-labelled with [^32^P]-ATP using T4 kinase (NEB). Competition with excess specific (unlabeled probes) and nonspecific competitors, a 593 bp internal coding sequence of *misR* obtained by PCR amplification using primer pair YT45 (5′-CGTAGATGACGATGCCCTGCTAACCG-3′) and YT46 (5′-GGCGGATGCTGGAGATGTGTACGTCG-3′), was performed to assess the specificity of the interaction.

### Capsule purification, electrophoresis and staining

Capsule polysaccharides were purified from strains NMB-CDC and MC58 as per Hobb *et al.* [22]. Capsule preparations were separated by deoxycholic acid (MP Biomedicals) polyacrylamide gel electrophoresis (DOC-PAGE) [65] and visualised by silver stain (Bio-Rad).

### Hydrophobic interaction chromatography (HIC)

The procedure for the hydrophobic interaction chromatography was essentially that of Tzeng *et al.* [31] and Karlyshev et al. [66]. Bacterial suspensions in PBS were loaded onto disposable plastic columns packed with 2 ml (bed volume) of octyl- Sepharose CL-4B resin (Sigma) and were washed with 5 ml of 0.2 M ammonium sulfate in 10 mM sodium phosphate buffer, pH 6.8. The proportion of bacteria cells that were eluted from the column was determined by optical density OD_600_.

### Microbial adhesion to solvents (MATS)

MATS was performed using the modified procedure of Ly et al. [67]. *N. meningitidis* was harvested from overnight growth on agar plates, resuspended in GC broth and centrifuged briefly at 60× g to remove clumps. The supernatant was measured and the cell suspension equalised to an OD_560_ of 0.4. The cells were pelleted by centrifugation at 3000× g and resuspended in 0.01 M potassium phosphate buffer (PPB). The pellet was washed once again and resuspended in PPB and the OD_560_ equilibrated to 0.4. Bacterial suspensions were mixed in a 6:1 ratio with hexadecane in glass McCartney bottles, vortexed for 30 sec to thoroughly mix the two phases and left to stand at room temperature for 30 min for the phases to separate. An aliquot of the aqueous phase was measured at an OD560 nm. The percentage hydrophobicity was calculated using the formula (1-(Abs2/Abs1))×100%.

## Results

### Genetic comparison of *N. meningitidis* strain NMB-CDC (ST-8 cc) with *N. meningitidis* strain MC58 (ST-32 cc)

*N. meningitidis* strain NMB-CDC is the prototype ST-8 strain belonging to ST-8 cc while strain MC58 is the prototype ST-32 strain belonging to ST-32 cc. We sequenced and annotated the genome of strain NMB-CDC and used orthologous group (OG) clustering (OrthoMCL) analysis to show that this isolate is highly related to G2136 from the ST-8 cc [6] and FAM18 prototype strain of ST-11 cc but was least conserved with ST-32 cc representative strain MC58 (Figure S1). Phylogenetic trees from previous studies (eg. Budroni et al. [6]) have also shown that ST-8 cc and ST-11 cc isolates are highly related to one another, and are more distantly related to strains from ST-32 cc. Table 3 summarises the alleles of putative virulence determinants possessed by strain NMB-CDC and MC58. Of interest, was the observation that strain NMB-CDC did not possess a phase variable *lgtA* indicating that expression of LNT-bearing LOS was not phase variable in this strain consistent with the sequenced genomes of strains from ST-11 cc (FAM18, M6190, ES14902, S0108 and K1207) and ST-8 cc (G2136 and 961-5945). In addition, both isolates had multiple copies of the meningococcal disease associated phage (MDA) (data not shown).

**Table 3:** A comparison of virulence encoding genes between strains NMB-CDC and MC58.

Protein	*Gene designation	Function	**Phase variable tract	
			MC58	NMB-CDC
LgtA	NMB1929	LNT expression [100]	Yes	No
LgtG	NMB2032	α2-3 LOS glycosyl-transferase [101]	Yes	No
+OpaA	NMB0442	Opacity protein/adhesin [102]	Yes	Yes
+OpaB	NMB1636	Opacity protein/adhesin [102]	Yes	Yes
+OpaD	NMB1465	Opacity protein/adhesin [102]	Yes	Yes
+OpaJ	NMB0926	Opacity protein/adhesin [102]	Yes	Yes
++Opc	NMB1053	Opacity protein/adhesin [16]	No	Absent
++PilC2	NMB0049	Pilus tip adhesion [12]	Yes	Absent
+++PilE	NMB0018	Major pilin [47]	No (I)	No (II)
PglA	NMB0218	Pilin glycan galactose transferase [82]	Yes	Yes
+++PglB	NMB1820	Pilin glycan synthesis [82]	No	No
PglE	NMB0624	Pilin glycan galactose transferase [82]	Yes	Yes
+++PglG	NMC0401	Pilin glycan glucose transferase [103]	Absent	Yes
+++PglH	NMC0400	Pilin glycan glucose transferase [103]	Absent	No
PglI	NMB1836	Pilin glycan O-acetyl transferase [104]	Yes	Yes
PptA	NMB0415	Pilin PEA transferase [105]	Yes	Yes
SynD	NMB0067	Sialic acid polymerase [106]	Yes	Yes
App	NMB1985	Neisserial penetration and adhesion protein [107]	No	No
MspA	NMB1998	Meningococcal serine protease A [108]	Yes	Absent
++++NadA	NMB1994	Neisserial adhesin A [109]	No (1)	No (3)
NhhA	NMB0992	Neisseria hsf/hia homologue [110]	No	No

* Gene designations as assigned to strain MC58 are NMBXXXX [111]. NMCXXXX designations for open reading frames found in strain FAM18 but absent in strain MC58 are used where appropriate [112].

** The potential for phase variation was determined by the length of the homopolymeric (>5 bp) or pentameric tracts (>5 copies) within these genes.

+ Strain MC58 has the following alleles: allele 96 (OpaA), allele 288 (OpaB), allele 147 (OpaD), allele 218 (OpaJ). The Opa loci from strain NMB-CDC were amplified and sequenced (see Methods) and were determined to be: allele 246 (OpaA), allele 277 (OpaB), allele 161 (OpaD) and allele 257 (OpaJ). This is the typical allelic pattern for ST-8 cc isolates [102].

++ Opc and PilC2 were absent from the sequenced genome of strain NMB-CDC and this was confirmed by PCR (see methods) [21].

+++ The sequence and expression profile of the pilin loci and glycosylation status of strain NMB-CDC has been published previously [34], [111]. (I) denotes Class I and (II) denotes Class II pilin expression [47].

++++ The allele designations of NadA are described in Comanducci et al. [109].

### Bacterial cell size differs between strains NMB-CDC and MC58

**Figure 1:**
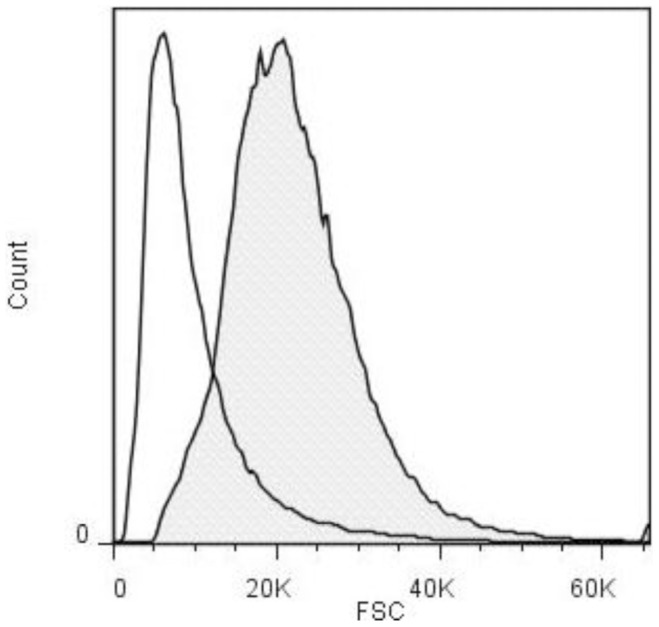
The surface area of strain NMB-CDC is larger than that of strain MC58. A representative flow cytometry histogram of the forward scatter (FSC) from strains MC58 (white area under the curve) and NMB-CDC (grey shaded area) analysed with the BD Influx cytometer. The mean of the FSC of NMB-CDC was 19,000 and the mean of the FSC of MC58 was 8,000 in this experiment.

While determining the viable counts for the attachment and invasion assays, it was noticed that at the same OD (OD_560_ of 0.4), the liquid cultures of strains NMB-CDC and MC58 yielded different viable counts. Strain NMB-CDC had 2.2+/−0.17×10^8^ colony forming units (cfu) per ml, whilst strain MC58 had a 5.5+/−38×10^8^ cfu/ml which is a 2.5-fold difference (p<0.0001). Since optical density correlates with biomass at mid-log phase [68], we postulated that as there is a direct proportional relationship between cell volume and mass, the cells of strain NMB-CDC must contain 2.5 times the dry mass and therefore have 2.5 times the volume of cells of strain MC58 [69]. If the relative volume of strain NMB-CDC is 2.5 and for strain MC58 is 1, then using a standard calculation for volume and surface area of a sphere, a single coccus of strain NMB-CDC was determined to possess a surface area 1.85 times larger than a single coccus of strain MC58. A comparison of the non-encapsulated variants of both strains, M7 and MC58Δ*synB*, using live samples and analysed by flow cytometry revealed that the surface area of strain M7 was 1.6-fold larger than that of MC58Δ*synB* (p<0.0001). Flow cytometry and high throughput microscopy using the AMNIS ImageStream^x^ confirmed the hypothesis that the cells of these isolates were of different sizes. Bacteria harvested from mid-log phase growth in liquid media, were fixed in 70% ethanol and then were analysed by AMNIS ImageStream^x^ which captures high resolution bright field and fluorescent images of the bacteria as they pass the detector. Fifty thousand events were captured and the in-focus images were gated into population R1 which was plotted according to scatter and bright field area (Figure S2). Examination of the images revealed that the sample could be divided into single diplococci (R2) and clumps (R3). Five hundred images were collected from each sample and the average pixel density was calculated for each sample set. Using this strategy, the surface area of the dehydrated cells of strain NMB-CDC was calculated to be 14.8 µm^2^ while MC58 had a surface area of 10.0 µm^2^, (p<0.0001), indicative of a 1.5-fold greater surface area for strain NMB-CDC. Lastly, live unfixed cells from the liquid mid-log phase cultures were processed by the BD Influx cell sorter with a small particle detector for analysis of bacterial cell size. The average forward scatter geometric mean of four independent experiments was calculated from 50,000 events. Since forward scatter is proportional to particle surface area [70] this data revealed that the cells of strain NMB-CDC had a surface area ∼1.89 fold greater than cells of strain MC58 (p<0.0001, Figure 1). In summary, two measurements of live unfixed and one made on fixed cells indicated that strain NMB-CDC had a surface area that was 1.85–1.89-fold and 1.5-fold greater, respectively, than strain MC58. To determine if bacterial cell size had an effect on growth in liquid culture, equivalent cfu/ml were used to inoculate GC broth and the optical density and number of cfu by viable count were examined at hourly time points until stationary phase. This experiment did not detect any difference in the rate of bacterial cell division under these conditions (data not shown).

### Strain NMB-CDC attaches to and invades human epithelial cells at significantly higher rates than strain MC58

**Figure 2:**
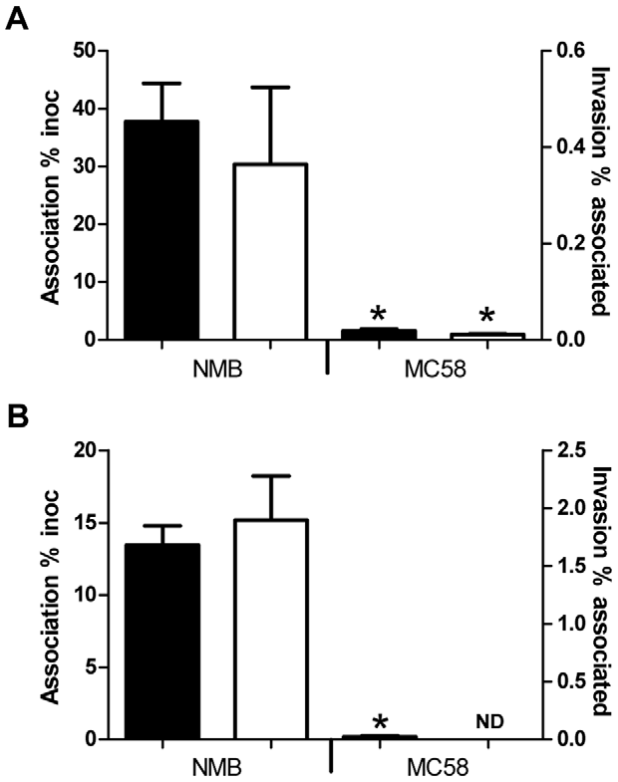
Strain NMB-CDC associates with and invades epithelial cell lines at greater rates than strain MC58. The rates of association and invasion of strains NMB-CDC and MC58 into Detroit 562 epithelial cells (Panel A) and human bronchial epithelial cells 16HBE14σ- (Panel B) were assessed. Attachment as percent of the inoculum (black bars read off the left y-axis) and invasion as the percentage of the associated bacteria (white bars read off the right y-axis) is shown. The average rate (+/–SEM) from three biological repeats in triplicate following 6 hrs co-incubation is shown. *: *p*<0.001 determined by Mann-Whitney t-test compared to strain NMB-CDC, ND: Not Detected, viable counts were below the limit of detection.

Since strain NMB-CDC and MC58 represent different genetic lineages and are different in bacterial cell surface area, we tested whether the strains interacted differently with host cells. The Detroit 562 pharyngeal carcinoma epithelial cell line and the transformed human bronchial epithelial cell line, 16HBE14σ- were challenged with suspensions of wild-type NMB-CDC and MC58 at a multiplicity of infection of 100. On Detroit 562 epithelial cells, 38% of the inoculum of strain NMB-CDC versus 1.5% of the inoculum of strain MC58 associated with the cells, respectively (p <0.0001, Figure 2A). Similar results were obtained for attachment to 16HBE14σ- with 13.5% of the inoculum of strain NMB-CDC versus 0.18% of strain MC58 associated with host cells (p = 0.0001, Figure 2B). Invasion was determined as the proportion of the adherent bacteria that were internalised at 6 hrs after the external bacteria were killed by treatment with gentamicin. Using this criterion, strain NMB-CDC invaded cells at significantly greater rates than MC58 in both cell lines. On Detroit 562 epithelial cells, 0.36% of attached NMB-CDC invaded and 0.01% of attached MC58 invaded (p = 0.0004, Figure 2A). Invasion by strain MC58 into 16HBE14σ- cells could not be determined as it was below the limit of detection. Invasion by strain NMB-CDC of 16HBE14σ- was 1.9% of inoculum (Figure 2B). To examine whether the adherent and invaded bacteria in these assays represented phase variants from the original inoculum with regards to capsule and LNT presentation, colony immunoblots were performed on the recovered isolates. In these assays the recovered colonies of NMB-CDC were found to have the same capsule and LOS phenotype as the inoculum, whereas a minor population of capsule and LOS phase variants were identified for strain MC58 as expected from previous studies (data not shown). Because invasion of MC58 into 16HBE14σ- cells could not be detected, the Detroit 562 cell line was used for the rest of the study.

### Type IV pili, regardless of class and glycosylation phenotype, are necessary for initial attachment by both strains to epithelial cells

**Figure 3:**
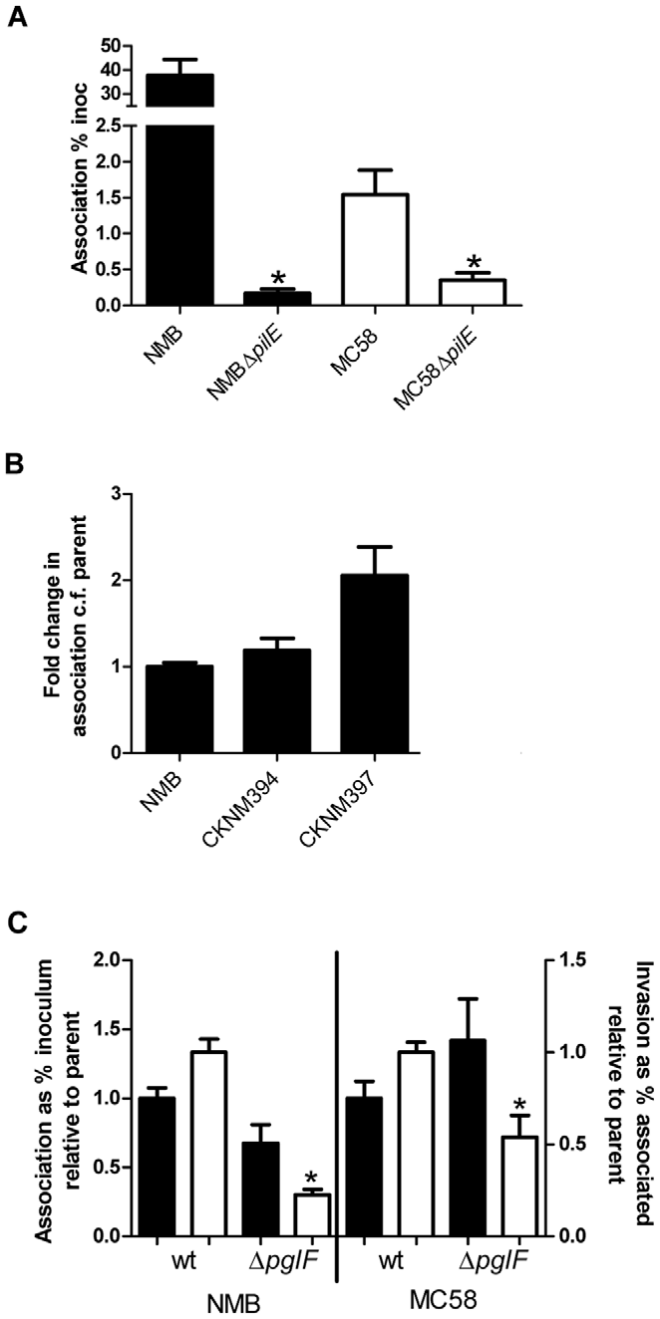
The role of Type IV pili is conserved in bacterial association with host cells. Panel A. The ability of strains lacking pili (Δ*pilE*) to attach to Detroit 562 cells was compared to the parental wild-type. Strains NMB-CDC and MC58 and their respective *pilE* mutant strains are shown in black and white, respectively. All strains were Opa+, Cap+ and LNT+. The average rate (+/−SEM) of association as a percentage of the inoculum of three biological repeats in triplicate as determined by viable counts following 6 hr co-incubation is shown. Panel B. The role of the class of pilin in association with host cells. Strain NMB-CDC was modified to express class I pilin from the *iga* locus in the absence of an intact *pilEII* locus (CKNM397). All strains were Opa+, Cap+ and LNT+. The ability of CKNM397 to associate with Detroit 562 cells was compared to parental wild-type strain NMB-CDC and CKNM394 containing an inactivated *iga* locus. The rate of association as a percentage of the inoculum of three biological repeats in triplicate was determined by viable counts following 1 hr co-incubation. The relative rate of association of each strain was normalised to strain NMB-CDC (value of 1) and is plotted as the average fold change (+/−SEM). *: *p*<0.005 determined by Mann-Whitney t-test. Panel C. The expression of a pilin glycan containing either GATDH (strain NMB-CDC) or DATDH (strain MC58) retains the same role in bacterial invasion for both strains of meningococci. The biosynthesis of the pilin glycan was interrupted by insertional inactivation of *pglF*. All strains were Pil+, Opa+, Cap+ and LNT+. The rate of association was determined by viable counts following 6 hr co-incubation. The relative rate of association of each strain was normalised to parental wild-type (value of 1) and is plotted as the average fold change (+/−SEM). Rates of attachment are shown in black bars (left y-axis) and invasion rates are shown in white bars (right y-axis). *: *p*<0.02 determined by Mann-Whitney t-test.

As strain NMB-CDC associated with Detroit 562 cells better than strain MC58, the role of the type IV pili which are of different classes was tested. Type IV pilin mutants of both NMB-CDC and MC58 (JKD5122 and CKNM417, Opa+, LNT+, Cap+, Pil−, Table 1) were constructed. These strains each adhered at a rate of 0.2% of the inoculum indicating that the type IV pilus was the critical determining factor for attachment of both strain NMB-CDC and MC58 to this cell line (p<0.0001 and p = 0.004 respectively, Figure 3A). As the rate of attachment of strains NMB-CDC and MC58 decreased from 38% and 1.5% respectively for the wild-type strains to 0.2% for their respective isogenic pilin mutants, we considered whether the differences in pilin class conferred different adhesive capabilities to the two strains. To determine whether the class of the pilin was contributing to the differences in host cell association between isolates, CKNM397 (Opa+, Cap+, LNT+, Table 1), which expresses the class I pilin from strain MC58 in a strain NMB PilEII-negative background, was constructed. There was no significant difference in the rate of association with host cells between CKNM397 and the control strain CKNM394 (Opa+, Cap+, LNT+, Pil+, Table 1), in which *iga* was insertionally inactivated (Δ*iga*::*aadA*) in the presence of PilEII+ (p = 0.053, Figure 3B). Both isolates express glycosylated pili, and the glycan structure has recently been shown by Jennings et al. [71] to initiate invasion into host cells by gonococci. To examine the role of the pilin glycan on attachment and invasion of strain NMB-CDC and strain MC58, a glycosylation (GLY) negative mutant (Δ*pglF*) was created in each strain in a Opa+, Pil+, LNT+, Cap+ background. Following a 6 hr incubation with Detroit 562 epithelial cells, no differences in attachment were observed when GLY was removed from the pili of either strain (Figure 3C). However, removal of the glycan resulted in a 4-fold (p = 0.0004) and 2-fold (p = 0.0078) decrease in invasion of these mutants compared to their respective parental strains NMB-CDC and MC58. Therefore, the pilin glycan did affect bacterial invasion, but to a similar extent in both isolates.

### Surface expressed sialic acid inhibits bacterial association and invasion of cell lines by strain MC58 to a greater extent than in strain NMB-CDC

Previous work has demonstrated that the expression of capsule and sialic acid (*N*-acetyl neuraminic acid, NANA) decorated LOS inhibits attachment and invasion of *N. meningitidis* into epithelial and endothelial cells [44]. Terminal sialylation of the LNT-bearing LOS also blocks Opa mediated invasion [48]. To determine the relative contribution of these structures to bacterial attachment and invasion, mutants in which the capsule was removed without affecting LOS sialylation (Δ*synD*, CAP-, NANA+) and mutants in which all surface sialic acid was removed (CAP-, NANA-), were constructed. All strains used in the following assays were Pil+, Opa+ and LNT+ by colony and western immunoblot (see Table 1).

**Figure 4:**
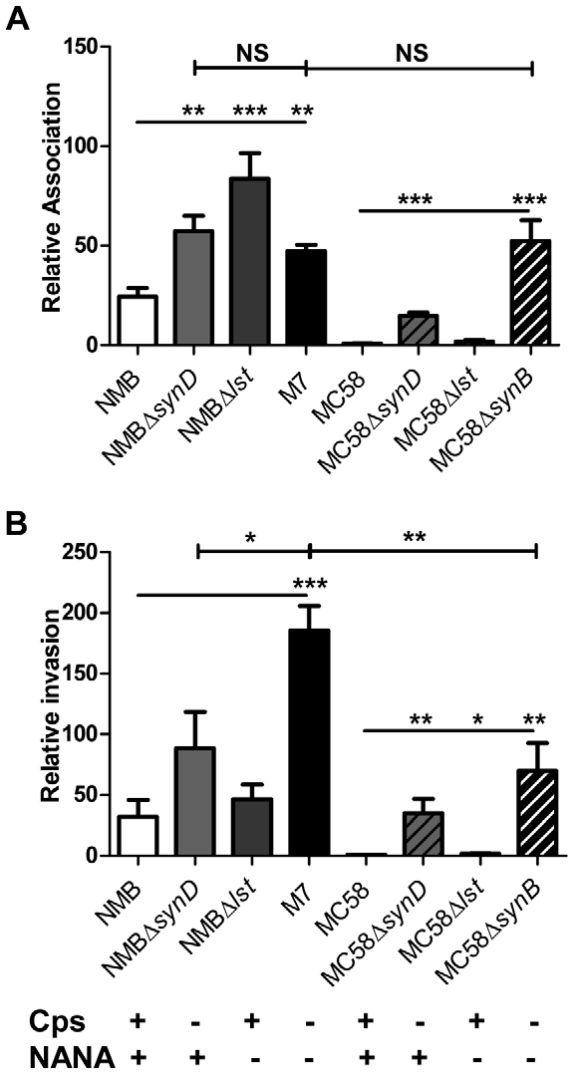
The effect of capsule and total sialic acid on attachment and invasion of strains NMB-CDC and MC58. Panel A and B show the rates of association and invasion, respectively, of capsule and sialic acid mutants relative to strain MC58. Parental wild-type (white bars), capsule mutants (Δ*synD*, light grey bars), LOS sialylation mutants (Δ*lst*, dark grey bars) and sialic acid mutants (Δ*synB*, black bars) in strain NMB-CDC (solid) and MC58 (diagonally stripes bars) are shown. The rate of association and invasion was determined by viable counts following 6 hr co-incubation. The relative rate of association of each strain was normalised to strain MC58 (value of 1) and values were plotted as the average fold change (+/−SEM). *: p<0.05, **:p<0.005, ***:p<0.0001 as determined by Mann-Whitney t-test).

The removal of capsule alone (Δ*synD* mutants, CAP-, NANA+) increased the rate of attachment of strain NMB-CDC by 2-fold and strain MC58 by 15-fold (p<0.002) (Figure 4A). The removal of capsule alone did not have a statistically significant effect on the invasion of strain NMB-CDC (p>0.2) however, the rate of invasion of strain MC58 increased 35-fold (p<0.0001) (Figure 4B). Eighty percent of the strain NMBΔ*synD* inoculum was associated with epithelial cells in this assay. The removal of LOS sialylation alone (Δ*lst* mutants, CAP+, NANA-) resulted in an increase in the rate of attachment (3.3-fold, p<0.0001) but not of invasion for strain NMB-CDC. In contrast, the rate of invasion increased slightly (1.7-fold, p = 0.034) but in the absence of a detectable change in attachment in the strain MC58 background. The removal of LOS sialylation in the absence of capsule (CAP-, NANA-) resulted in no further gains in the rate of attachment of strain NMB-CDC to Detroit cells. However, the rate of invasion of NMB-CDC without sialic acid increased by 2.1-fold when compared to absence of capsule alone (p = 0.028) and 6-fold when compared to wild-type NMB-CDC (p<0.0001). In contrast, the removal of LOS sialylation in the absence of capsule (CAP−, NANA−) in the strain MC58 background conferred a further 3.5-fold increase in attachment (p<0.0001) but no change in invasion (p = 0.49) which is a 52-fold increase in attachment and 70-fold increase in invasion compared to wild-type MC58 (p<0.005).

Importantly, there was no significant difference in the attachment of MC58Δ*synB* compared to M7, indicating parity between the strains was achievable when sialic acid biosynthesis was ablated, despite the multitude of variable protein alleles between the two strains. The difference in the rate of invasion of strains NMB-CDC and MC58 decreased from 30-fold in the presence of sialic acid to only 2.6-fold in the absence of capsule indicating the surface sialic acid had a major influence on this process but was not the only contributing factor to the invasion rate.

### Strain NMB-CDC and strain MC58 express the same amount of surface capsule

**Figure 5:**
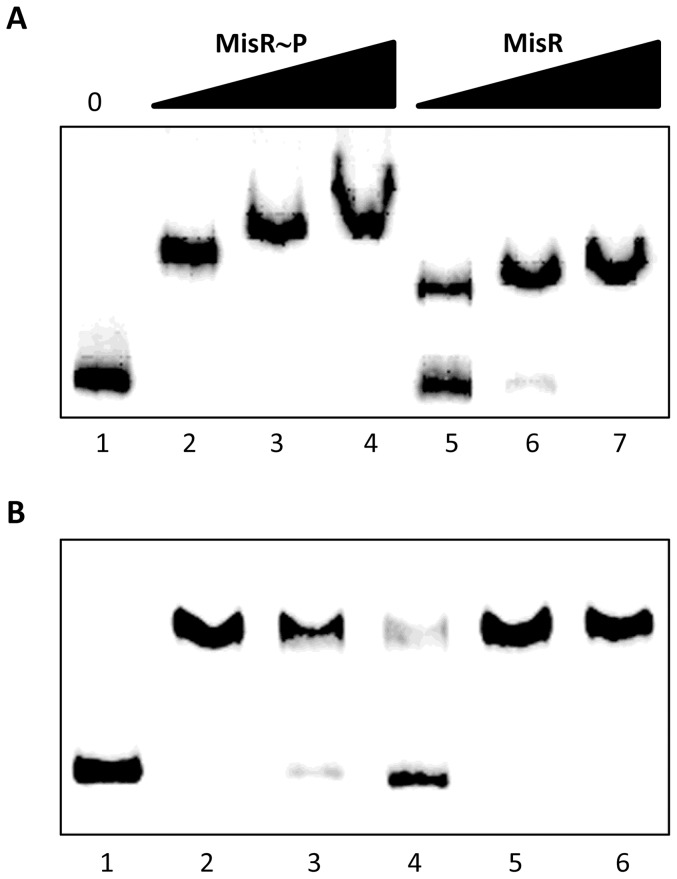
MisR directly binds to the *synA-ctrA* promoter region. Panel A. MisR interacts with the *cps* intergenic probe containing both the *synA* and *ctrA* promoters. Phoshorylated MisR (MisR∼P, Lanes 2–4) and MisR (lanes 5–7) binds the probe in a dose-dependent manner. Lane 1 contains probe alone, Lanes 2 and 5 contain 68 pmol of protein; lanes 3 and 6 contain 136 pmol of protein; lanes 4 and 7 contain 204 pmol of protein. Panel B. Competition EMSA. The mobility shift of the labeled probe by MisR∼P (136 pmol, lane 2) relative to labeled probe without protein (lane 1) was competed away by unlabelled probe (1 µg in lane 3 and 2 µg in lane 4). This interaction between MisR∼P and the labeled probe was specific as the complex remained intact and could not be competed away when cold, unlabeled non-specific DNA was added (1 µg in lane 5 and 2 µg in lane 6).

As the capsule of strain MC58 inhibited attachment and invasion to a greater extent than the capsule of NMB, we examined capsule expression in the two isolates. In serogroup B meningococci, capsule is synthesised via the *synABCD* locus which is divergently transcribed from an intergenic promoter region with the capsule transport locus of *ctrABCD* [72] and the entire region is termed the *cps* locus. The divergent promoter region in the *cps* locus is identical between strain NMB-CDC and MC58, and no IS1301 element was present (data not shown). We have previously shown that the two-component response regulator MisR represses the *cps* locus and de-repression by mutation of this regulator also results in hyper-encapsulation and serum resistance in strain NMB-CDC [61]. To test whether MisR (which was identical in the two isolates) regulated the *cps* locus through a direct interaction with the intergenic promoter region, an EMSA was conducted (Figure 5A). MisR interacted with the *cps* intergenic probe containing both the *synA* and *ctrA* promoters in a dose-dependent manner and phosphorylated MisR generated by acetyl phosphate incubation showed a higher affinity (compare lanes 2 and 5, Figure 5A). Further, the competition EMSA confirmed that this interaction was specific (Figure 5B). The transcription of both *synA* and *ctrA* increased in the *misR* mutants of both strains compared to the parental isolates as determined by qRT-PCR (Table 4). There were no significant differences in the levels of transcription of either *synA* or *ctrA* between the paired parental isolates or the paired Δ*misR* mutants. The expression of *lgtG* and *dsbD* both of which are regulated by MisR [29], [73] and of *lst*, which is not part of the MisR regulon, were also consistent between the two isolates.

**Table 4:** qRT-PCR of gene expression in strains NMB-CDC and MC58 and their respective Δ*misR* mutants.

Strain	Gene	wild-type		Δ*misR*		Fold change	p-value
		Average/*lpxA*	st. dev.	Average/*lpxA*	st. dev.		
NMB	*lst*	1.79	0.25	2.33	1.09	1.3	0.4879
	*ctrA*	4.48	0.84	17.30	5.37	3.9	0.0016
	*synA*	4.27	2.35	8.17	1.57	1.9	0.0130
	*lgtG*	0.05	0.03	0.68	0.32	13.6	0.0051
	*dsbD*	10.94	2.04	1.41	0.25	0.13	3×10^−6^
MC58	*lst*	1.73	0.76	3.10	0.71	1.8	0.1322
	*ctrA*	5.49	2.76	20.86	7.14	3.8	0.0020
	*synA*	5.16	2.31	11.47	4.13	2.2	0.0176
	*lgtG*	0.10	0.06	1.09	0.57	10.9	0.0046
	*dsbD*	15.10	7.09	2.29	1.04	0.15	0.0040

**Figure 6:**
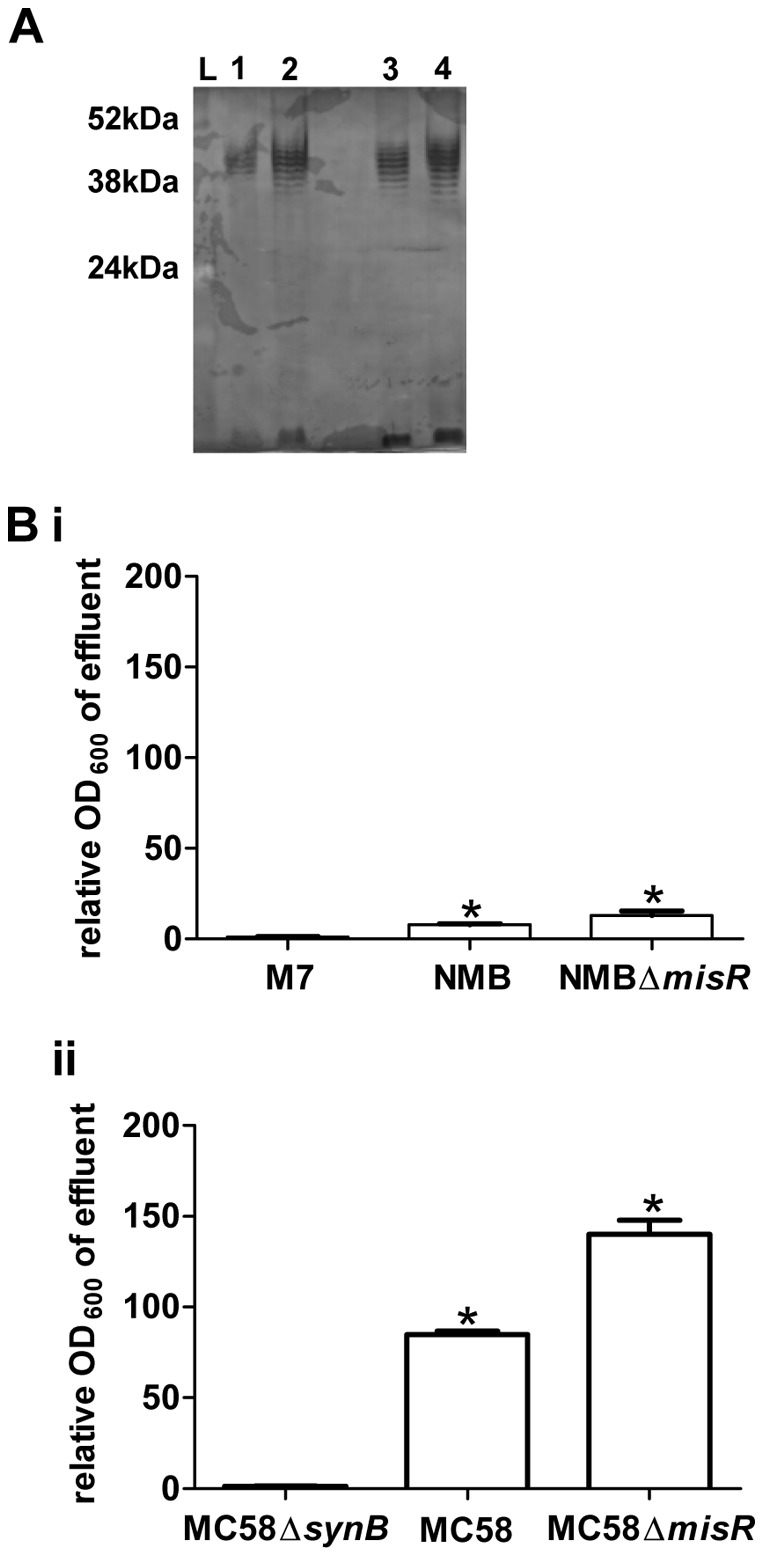
Analysis of the capsule polymer and surface hydrophobicity profiles of strains NMB-CDC and MC58. Panel A. Physical modality and length of the capsule polymers of both strains are the same. Purified capsule preparations (5 µg [lanes 1 and 3] and 10 µg [lanes 2 and 4]) from strains MC58 (lanes 1 and 2) and NMB (lanes 3 and 4) were separated by DOC-PAGE and silver stained. Panel B. Comparison of surface hydrophobicity of strains NMB-CDC and MC58 using HIC. Surface hydrophobicity of strains NMB-CDC (A) and MC58 (B) with their respective isogenic mutants (non-encapsulated, M7 and MC58Δ*synB* and hyper-encapsulated variants, NMBΔ*misR* and MC58Δ*misR*) were assessed by interaction chromatography. The proportion of bacteria eluted from the column was determined by optical density and is presented relative to the isogenic non-encapsulated control (M7 for NMB and MC58Δ*synB* for MC58). The average of at least three biological repeats (+/−SEM) for each strain is shown. *: *p*<0.01 determined by unpaired t-test compared to the non-encapsulated controls.

We examined the length of the capsule polymers of NMB-CDC and MC58 by separation of purified capsule by DOC-PAGE visualised by silver-staining. With the resolution of this analysis, it appears that both strains express a capsule polymer of similar length and modality (Figure 6A) [74]. The capsule of NMB-CDC and MC58 was examined by flow cytometry following labeling with anti-capsule antibody and this revealed that the geometric mean fluorescence (GMF) of strains NMB-CDC and MC58 were 24.03 and 20.76, respectively. Based on the transcriptional data and the quantitation of capsule polymer by FACs, it appears both strains express similar amounts of surface capsule.

### The surface of strain NMB-CDC is more hydrophobic than strain MC58

Previous studies have shown that hydrophobicity of bacterial surfaces is a function of capsule expression [31], [75], [76], [77] and we reasoned that if there was a difference in capsule distribution on the bacterial surface of these two isolates this would be detected using hydrophobicity interaction chromatography (HIC, [31]) and microbial adhesion to solvents (MATS) assays. When using HIC, encapsulated isolates are more hydrophilic than non-encapsulated isogenic mutants, and thus the encapsulated bacteria will pass through the column in the effluent [31]. The non-encapsulated control strains M7 and MC58Δ*synB* were almost completely retained on the column and were not significantly different (p = 0.06), indicating that the capacity of the column to bind the bacterial cells had not been saturated (Figure 6B). For strain NMB-CDC the OD of the effluent was 8-fold higher than that for M7 (p<0.0001). In comparison, the effluent of MC58 was 85-fold higher than that of MC58Δ*synB* (p<0.0001). Since the effect of the loss of surface sialic acid from strain NMB-CDC was 10-fold less than that observed for strain MC58 using these columns, the surface of strain NMB-CDC was likely to be more hydrophobic than strain MC58. To further confirm that the hydrophobicity interaction columns could distinguish between hydrophilic surfaces with more or less capsule polymer, the effect of capsule over-expression was examined. A Δ*misR* mutant of strain NMB-CDC was shown previously to express ∼40% more capsule polymers than the parent strain, which led to an increase in resistance to normal human serum thus indicating that the capsule was on the bacterial cell surface [61]. The OD of the effluent for the NMBΔ*misR* and MC58Δ*misR* mutants was 1.7-fold higher than their respective parents (p<0.001), consistent with the increased surface expression of capsule in the mutant strains and hence a decrease in surface hydrophobicity.

Since HIC was considered to be a qualitative assay, MATS was used to provide a quantitative measure of hydrophobicity for these isolates. In MATS, the level of adhesion of the bacteria to non-polar *n*-alkane, hexadecane, is measured and the percentage of the adhesion to the solvent (% hydrophobicity) is determined [78]. In this assay, the non-encapsulated strains NMB Δ*synA* and MC58Δ*synB* were 2.2-fold (p = 0.004) and 4.1-fold (p = 0.0002), than their respective parental encapsulated wild-type strains (Table 5). Although the hyper-encapsulated NMBΔ*misR* mutant was 2.16 fold less hydrophobic than the parental wild-type (p = 0.005), no difference between strain MC58 and MC58Δ*misR* was observed (p>0.1). No further decrease in hydrophobicity of the hyper-encapsulated MC58Δ*misR* could be detected in buffers of increasing ionic strength (data not shown, [78]), suggesting that strain MC58 was already very hydrophilic and no further reductions in hydrophobicity could be measured in this assay. Overall, both the HIC and MATS assays correlated closely with each other and indicated that encapsulated strain NMB-CDC was more hydrophobic than encapsulated strain MC58.

**Table 5:** Adhesion of bacterial strains to hexadecane in the MATS assay.

Strain	Percentage hydrophobicity (Mean±standard deviation)*
NMB	27.7±3.7
NMBΔ*synA*	62±9.5
NMBΔ*misR*	12.8±2.9
MC58	10.8±3.0
MC58Δ*synB*	41.5±7.0
MC58ΔmisR	6.8±3.5

* The mean of three biological repeats each containing three technical repeats.

## Discussion

Models investigating key steps in the pathogenesis of meningococcal attachment and invasion of human cells, have shown the importance of phase variable expression of pili, capsule, LOS, Opa and Opc [12], [15], [16], [19], [33], [37], [38], [39], [40], [41], [42], [43], [44], [51], [79], [80]. We hypothesised that since strain NMB-CDC has a significantly lower phase variation frequency and was unable to phase vary the LNT-bearing LOS to relieve inhibition of Opa adhesins, attachment and invasion of human cells would be comparatively less than strain MC58. Surprisingly, an encapsulated, LNT-bearing LOS, class II piliated and Opa expressing strain NMB-CDC was much more efficient than the encapsulated, LNT-bearing, class I piliated and Opa expressing strain MC58 in attachment and invasion on two model cell lines (Figure 2). The association and invasion data for strain MC58 derived from this model cell line is comparable with the outcomes described for this strain in previous work with other epithelial and endothelial cell lines [15], [16], [19], [39], [40], [41].

The current hypothesis generated from an examination of the genomes of non-disease and disease causing isolates of meningococci, predicts that differences in the combination of alleles of virulence factors, including type IV pili, capsule, and LOS structure, would mediate different interactions with the host cell. To test this hypothesis, the effect of pilin class and glycosylation, sialylation of LNT-bearing LOS and capsule expression by both strains was examined in greater detail. ST-8 cc strain NMB-CDC expresses a typical class II type IV pilus in comparison to ST-32 cc strain MC58 which expresses a class I variant [34]. In both isolates the type IV pili had the same predominant effect on bacterial attachment to host cells (Figure 3). Exchanging the expression of class II pili to class I pili in strain NMB-CDC did not affect the performance of strain NMB-CDC to attach to host cells, indicating that the class of the pilin had no discriminatory effect in this process. Meningococcal type IV pili are variably glycosylated with a tri-saccharide [81]. The glycan generally consists of two galactose residues linked to 2,4-***d***i***a***cetamido-2,4,6-***t***ri***d***eoxy***h***exose (DATDH) [82], although a variant glycan containing ***g***lyceramido ***a***cetamido ***t***ri***d***eoxy***h***exose (GATDH) is produced by strains containing a variant synthesis gene, *pglB2* [34], [83]. A similar glycan is present on class I type IV pili of gonococci and has been shown by Jennings et al. [71] to interact with complement receptor 3 to initiate invasion into *pex* cells. While both strain NMB-CDC and MC58 produce glycosylated pili, strain NMB-CDC expresses the glycan containing GATDH and strain MC58 expresses a glycan containing DATDH. Nevertheless, the removal of the glycan by inactivation of the synthesis gene, *pglF*, in both strains resulted in a decrease in rate of invasion without affecting bacterial association to host cells [10], [84]. Lastly, the type IV pili contain antigenically variable PilC tip adhesins [85] which previous studies have implicated in tissue tropism. However since the differences in the ability of strain NMB-CDC and MC58 to attach to Detroit 562 cells reached parity when the amount of surface sialic acid was modulated on the bacteria, it was concluded that no significant differences in attachment could be attributed to the PilC alleles expressed by strains NMB-CDC and MC58 under the experimental conditions used in this study. Therefore, the enhanced performance of strain NMB-CDC to invade host cells when compared to strain MC58 was not attributable to the variant type IV pili expressed by these two isolates.

Previous work has clearly identified the capsule and terminal sialic acid of LNT-bearing LOS as two components which interfere with Opa function and bacterial attachment to host cells [19], [48]. In strain MC58, the inhibitory effect of sialylated LNT-bearing LOS is relieved by the phase variable removal of the LNT moiety [19]. However, since strain NMB-CDC cannot phase vary the expression of LNT as it lacks a phase variable *lgtA*, the role of LOS sialylation was examined in the presence of LNT-bearing LOS with and without capsule. In agreement with previous studies with strain MC58, capsule was the major determinant of inhibition of bacterial attachment and invasion of host cells while removal of sialylation of LNT-bearing LOS resulted in no change in bacterial association or invasion unless capsule was also absent [19] (Figure 4). In contrast, in strain NMB-CDC, removal of sialylation of LNT-bearing LOS increased rates of attachment by 2-fold indicating that LOS itself could affect invasion even in the presence of capsule in this strain. Removal of capsule from strain NMB-CDC expressing LNT-bearing LOS resulted in only a 3-fold increase in bacterial association suggesting that both LOS sialylation and capsule had equivalent inhibitory roles, rather than the predominant effect of capsule alone that was seen in strain MC58 (Figure 4). Therefore, these data indicated that the serogroup B capsule of strain NMB-CDC was much less inhibitory to bacterial attachment and invasion than observed for strain MC58.

A number of theories were tested regarding the potential for variation in capsule presentation on the surface of these two isolates. The MisR-dependent regulation of capsule expression, and the measurement of surface capsule by flow cytometry, in addition to the length and modality of the polymers was similar for both strains (Table 4). Since meningococcal capsule is a glycolipid anchored to the surface of the bacterial outer membrane, it has a distribution profile per unit surface area. Therefore, as strain NMB-CDC had a larger surface area than strain MC58, the distribution of the anchored glycolipid polymer on the bacterial cell surface of NMB-CDC was hypothesised to be sparser than that of strain MC58. Capsule polysaccharide is a major determinant of surface hydrophobicity in numerous bacteria [66], [75], [76], [77] due to the ability to retain polar water molecules close to the bacterial surface. The capsule polymer of strain NMB-CDC contributed to a smaller change in surface hydrophobicity (8-fold by HIC, and 2.2-fold by MATS) than in strain MC58 (85-fold by HIC and 4.4-fold by MATS), indicating that encapsulated strain NMB-CDC was more hydrophobic than strain MC58. Our model suggests that as the capsule polymers become sparser over a greater surface area, the overall surface of the isolate becomes more hydrophobic. However, surface hydrophobicity is a multi-factorial phenotype, resulting from the complex interplay of polar and apolar outer surface components, including major surface proteins in some bacterial species [86], [87], [88] and O-antigen of lipopolysaccharide [89]. Future work will address whether other factors contribute to surface hydrophobicity in the presence of capsule in meningococci.

Lastly, bacterial cell size has been shown to be controlled by a number of factors including nutrient availability [90] and cell cycle control by the initiation of replication protein, DnaA [91]. Weart et al. [90] identified a metabolic sensor, UgtP, which localised at the cell division site in a nutrient dependent manner, inhibiting cell division and thus controlling cell size in the rod-shaped organism *Bacillus sp*. However, a search of the meningococcal genome did not find a homologue of this protein, suggesting other factors are needed for this phenotype in cocci. It is important to note that the Family *Neisseriaceae* contains both pathogenic and commensal bacterial species of variable cell dimensions encompassing diplococci, coccobacilli and rods [92]. The difference in bacterial cell size between these two isolates suggests that their metabolism is different. Multiple studies of the conserved and accessory genomes of meningococcal isolates [93] and variation within the housekeeping genes used for multi-locus sequence typing [94], has recently led to the proposal that small changes in metabolism may lead to differences in virulence and transmission of isolates.

In conclusion, the two classes of type IV pili and pilin glycosylation with glycans containing either GATDH or DATDH, were shown to have conserved roles during attachment and invasion into host cells by the two exemplar meningococcal isolates in this study. The surface of encapsulated strain MC58 was shown to be more hydrophilic and adhered less to host cells than strain NMB-CDC. Improved levels of adherence of strain NMB-CDC to cell lines correlated with increased bacterial cell surface and surface hydrophobicity. At this stage, future work will be required to understand the genetic and metabolic basis for the variability of meningococcal bacterial cell size and surface hydrophobicity, and to determine whether these phenotypes are shared by other isolates from the same ST or clonal complex.

## Supporting Information

Figure S1 **Venn diagram showing the distribution of genes as orthologous groups (OG) across four genomes representing ST-8 cc (NMB-CDC, G2136), ST-11 cc (FAM18) and ST-32 cc (MC58).** Orthologous group (OG) clustering of open reading frames using a cut-off of 1e -50 to identify new genes in strain NMB-CDC versus those of G2136, FAM18 and MC58. Strain NMB-CDC most closely aligns with the ST-8 cc genome of strain G2136 with which it shares 1834 genes. In addition, strain NMB-CDC is more distantly related to strain MC58 with which it shares 1659 genes. The core or shared genome of the four isolates in this collection consists of 1579 genes. Strain MC58 from ST-32 cc was the most divergent from the other three genomes having 242 genes unique to this isolate.
(TIF)

Figure S2 **AMNIS ImageStream^x^ can be used to identify single diplococci in an NMB-CDC sample under flow.** Panel A: Bivariate plot of the bright field and scatter channels for the particles in focus (R1 focus). Panel B. Image gallery of hand tagged cells for analysis from the R2 region with bright field images displayed on the left and corresponding SYTOX Green image on the right. Panel C: Image gallery of particles in the R3 region with brightfield images displayed on the left and corresponding SYTOX Green image on the right. The example given here is for strain NMB, but the same analysis was performed for preparations of strain MC58 (not shown).
(TIF)
