# Supplementary figures and images for: Attachment and Invasion of Neisseria meningitidis to Host Cells Is Related to Surface Hydrophobicity, Bacterial Cell Size and Capsule
Source: PLoS One. 2013 Feb 6;8(2):e55798. doi: 10.1371/journal.pone.0055798 (PMC3566031; doi:10.1371/journal.pone.0055798)

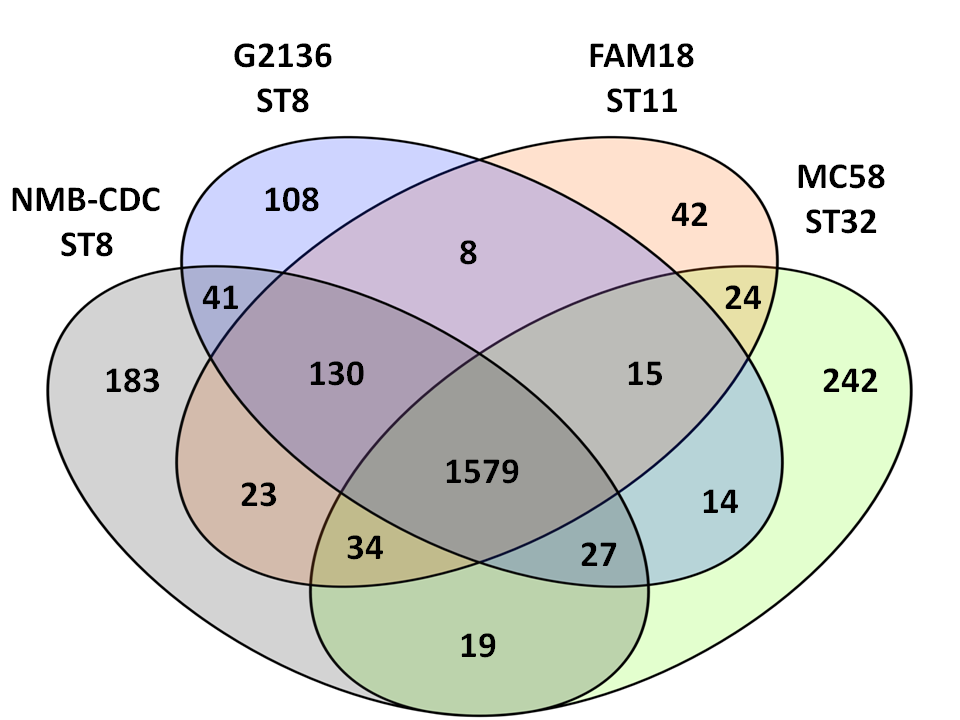

Supplement: Figure S1 — Venn diagram showing the distribution of genes as orthologous groups (OG) across four genomes representing ST-8 cc (NMB-CDC, G2136), ST-11 cc (FAM18) and ST-32 cc (MC58). Orthologous group (OG) clustering of open reading frames using a cut-off of 1e -50 to identify new genes in strain NMB-CDC versus those of G2136, FAM18 and MC58. Strain NMB-CDC most closely aligns with the ST-8 cc genome of strain G2136 with which it shares 1834 genes. In addition, strain NMB-CDC is more distantly related to strain MC58 with which it shares 1659 genes. The core or shared genome of the four isolates in this collection consists of 1579 genes. Strain MC58 from ST-32 cc was the most divergent from the other three genomes having 242 genes unique to this isolate. (TIF) [file pone.0055798.s001.tif]

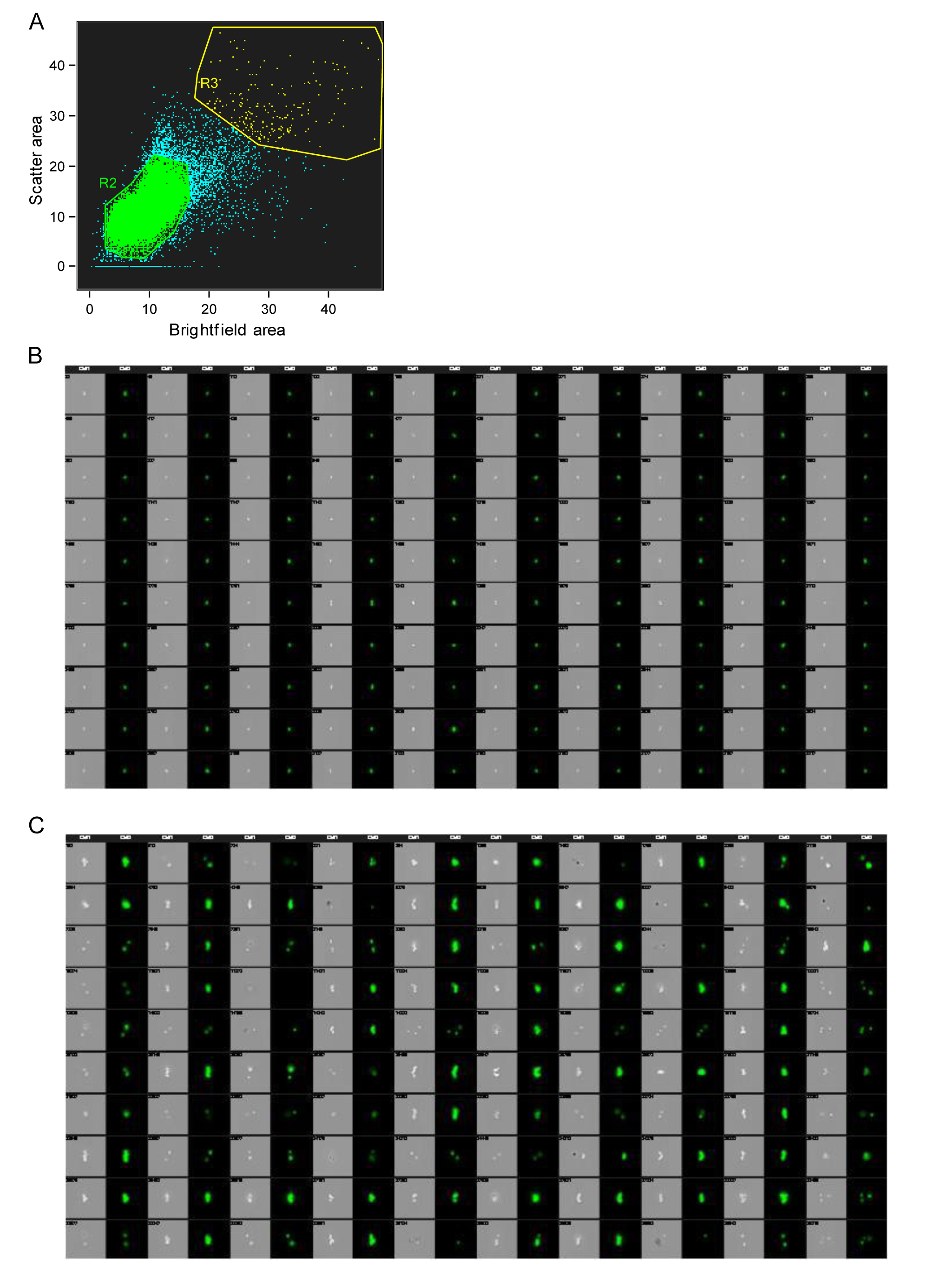

Supplement: Figure S2 — AMNIS ImageStreamx can be used to identify single diplococci in an NMB-CDC sample under flow. Panel A: Bivariate plot of the bright field and scatter channels for the particles in focus (R1 focus). Panel B. Image gallery of hand tagged cells for analysis from the R2 region with bright field images displayed on the left and corresponding SYTOX Green image on the right. Panel C: Image gallery of particles in the R3 region with brightfield images displayed on the left and corresponding SYTOX Green image on the right. The example given here is for strain NMB, but the same analysis was performed for preparations of strain MC58 (not shown). (TIF) [file pone.0055798.s002.tif]
